# Circulating tumour cells escape from EpCAM-based detection due to epithelial-to-mesenchymal transition

**DOI:** 10.1186/1471-2407-12-178

**Published:** 2012-05-16

**Authors:** Tobias M Gorges, Ingeborg Tinhofer, Michael Drosch, Lars Röse, Thomas M Zollner, Thomas Krahn

**Affiliations:** 1 Bayer Pharma AG, Muellerstr. 178, Berlin 13353, Germany; 2 Charite CCM, Klinik für Radioonkologie und Strahlentherapie Chariteplatz 1, Berlin 10117, Germany; 3 Boehringer Ingelheim Pharma GmbH & Co. KG Drug Metabolism&Pharmacokinetics, Biberach, Germany

**Keywords:** Circulating tumour cells, Breast cancer, Xenograft, Metastasis, Epithelial-mesenchymal transition

## Abstract

**Background:**

Circulating tumour cells (CTCs) have shown prognostic relevance in metastatic breast, prostate, colon and pancreatic cancer. For further development of CTCs as a biomarker, we compared the performance of different protocols for CTC detection in murine breast cancer xenograft models (MDA-MB-231, MDA-MB-468 and KPL-4). Blood samples were taken from tumour bearing animals (20 to 200 mm^2^) and analysed for CTCs using 1. an epithelial marker based enrichment method (AdnaTest), 2. an antibody independent technique, targeting human gene transcripts (qualitative PCR), and 3. an antibody-independent approach, targeting human DNA-sequences (quantitative PCR). Further, gene expression changes associated with epithelial-to-mesenchymal transition (EMT) were determined with an EMT-specific PCR assay.

**Methods:**

We used the commercially available Adna Test, RT-PCR on human housekeeping genes and a PCR on AluJ sequences to detect CTCs in xenografts models. Phenotypic changes in CTCs were tested with the commercially available “Human Epithelial to Mesenchymal Transition RT-Profiler PCR Array”.

**Results:**

Although the AdnaTest detects as few as 1 tumour cell in 1 ml of mouse blood spiking experiments, no CTCs were detectable with this approach in vivo despite visible metastasis formation. The presence of CTCs could, however, be demonstrated by PCR targeting human transcripts or DNA-sequences - without epithelial pre-enrichment. The failure of CTC detection by the AdnaTest resulted from downregulation of EpCAM, whereas mesenchymal markers like Twist and EGFR were upregulated on CTCs. Such a change in the expression profile during metastatic spread of tumour cells has already been reported and was linked to a biological program termed epithelial-mesenchymal transition (EMT).

**Conclusions:**

The use of EpCAM-based enrichment techniques leads to the failure to detect CTC populations that have undergone EMT. Our findings may explain clinical results where low CTC numbers have been reported even in patients with late metastatic cancers. These results are a starting point for the identification of new markers for detection or capture of CTCs, including the mesenchymal-like subpopulations.

## Background

Metastasis is a multistep process in the course of which tumour cells migrate into the circulation, generating circulating tumour cells (CTCs) in the blood stream or disseminated tumour cells (DTCs) that home to the bone marrow [1]. It is thought that epithelial tumour cells have to undergo phenotypic changes to overcome the successive barriers to intravasation, survival in the blood, extravasation and secondary tumour formation [2]. These changes are accompanied by a reduction of cell-cell adhesion, loss of apical-basolateral polarity, and loss of epithelial marker expression whereas the expression of mesenchymal-associated genes is induced [3]. This developmental program, described as epithelial-to-mesenchymal transition (EMT), may be a prerequisite for invasive growth and metastatic spread [4]. Although the role of EMT for CTCs is still under debate, recent findings support the hypothesis that CTC profile changes occur during tumour cell dissemination [5-7]. As EMT processes in human samples are hard to follow and because of the difficulty of detecting CTCs in humans [8], animal models could help to evaluate and dissect the functional implications of EMT specific processes in vivo.

CTCs could already be detected in blood samples of an orthotopic breast cancer model (MDA-MB-231) using a modified version of the FDA approved CellSearch technique [9]. In xenograft models of prostate cancer, however, CTCs could only be detected after a second round of tumour cell inoculation after one in vivo passage [10]. CTCs isolated from MDA-MB-231 breast cancer xenografts display a more aggressive phenotype than the original cells, indicating the impact of CTCs for metastatic formation [11]. In addition, phenotypic changes on CTCs in animal models were also demonstrated [12], showing that CTCs are technically suitable for functional characterisation. We compared different breast cancer models (MDA-MB-231, MDA-MB-468 and KPL-4) in order to test their suitability to serve as model system for CTC formation. The fast emergence of mesenchymal-related CTC populations led to identification of new markers for CTC detection.

## Methods

### Cell culture

Human breast cancer cell lines MDA-MB-231 (ECACC, #92020424), MDA-MB-468 (ATCC, #HTB-132) and KPL-4 (Kawasaki Medical School) were cultured in DMEM/F12 + 200 mM glutamine (MDA-MB-231, MDA-MB-468) or RPMI + Glutamax (KPL-4) supplemented with 10% fetal calf serum (FCS) and 1% penicillin/streptomycin. Cells were maintained at 37°C in a humidified incubator with 5% CO_2_. Adherent cells were harvested at 80% confluency by washing with sterile Dulbecco’s phosphate buffered saline (PBS) followed by trypsinisation for 5 min at 37°C.

### Western blot

For the detection of EpCAM (epithelial cell adhesion molecule) and MUC-1 (mucin-1) expression, Western Blot analysis was performed. 20 μg of total cell protein were separated using SDS polyacrylamide gel electrophoresis (SDS-PAGE), and proteins were transferred to a nitrocellulose membrane. The membrane was blocked with PBS containing 0.1% Tween-20 (PBS-T) and 5% skimmed milk powder for 1 h at room temperature (RT). Following the blocking step, the EpCAM (abcam; [E144] (ab32392), 1 : 100) and MUC-1 (abcam; [EP1024Y] (ab45167), 1 : 1,000) primary antibodies were added and incubated over night at 4°C. The blot was washed three times with PBS-T. Next, horseradish peroxidase-conjugated secondary antibody was added to the blocking solution (GE Healthcare: NIF824, 1 : 5,000) and incubated for 2 h at RT. After three washing steps with PBS-T, detection was carried out using Amersham ECL Western Blotting Systems (RPN2232) and Imagequant software. Tubulin (Sigma: T6074, 1:2000) detection was included as loading control. The secondary antibody was purchased from GE Healthcare (NA93IVS).

### Flow cytometry

5 x 10^5^ cells were detached by trypsinisation, washed and stained for 30 min at RT in the dark. For the analysis of EpCAM expression tumour cells were stained with an APC-labeled (allophycocyanin) anti-EpCAM antibody (BD Bioscience, Cat. no. 347200; 1:200). FACS (fluorescence activated cell sorting) analysis was performed (FACScalibur) with addition of SYTOX® Red Dead Cell Stain (Invitrogen, Cat. No. S34859) to exclude dead cells. 10,000 cells were counted for the analysis.

### AdnaTest system

The AdnaTest System is a method based on immunomagnetic enrichment of epithelial tumour cells using antibodies against EpCAM and MUC-1 followed by mRNA isolation via oligo(dT)-conjugated magnetic particles for the analysis of tumour associated gene expression by RT-PCR (EpCAM, MUC-1, Her2 (human epithelial growth factor receptor 2), and Actin). This assay was developed for the detection of CTCs in 5 ml of human blood. Since much smaller amounts of blood can be collected from murine models (800 to 1,000 μl, or ~1/5 of the recommended blood volume), we reduced the BreastSelect Beads (BreastCancerSelect, Article no. T-1-508) to 25 μl instead of 100 μl per sample. The rest of the protocol was carried out according to the manufacturer’s instructions. In the PCR reaction (run on a Veriti Thermal Cycler, Applied Biosystems), amplicons of the following sizes were generated: EpCAM: 395 base pairs (bp), MUC-1: 293 bp, Her2: 270 bp, and Actin: 114 bp. Visualisation of the PCR products was carried out with a 2100 Bioanalyser using DNA 1000 LabChips (Agilent Technologies), as recommended by AdnaGen.

### Qualitative CTC detection - targeting human gene transcripts

100 μl blood samples were collected from mice in EDTA-tubes (S-Monovette, Sarstedt) and transferred to RNAprotect Animal Blood Tubes (Qiagen Cat. No. 76544) for immediate stabilisation of cellular RNA. Next, total RNA was purified using the RNeasy Protect Animal Blood Kit (Qiagen, Cat. No. 73224). cDNA synthesis was performed with 800 ng of RNA using the High Capacity RNA-to-cDNA Kit from Applied Biosystems (Cat. No. 4387406). For CTC detection, we designed human-specific primers targeting mRNA of the housekeeping genes glycerinaldehyd-3-phosphat-dehydrogenase (GAPDH) [*Fwd 5`-ATCATCCCTGCCTCTACTGG-3`/Rev 5`-GTCAGGTCCACCACTGACAC-3`*] and peptidylprolyl isomerase A (PPIA) [*Fwd 5`-TTCATCTGCACTGCCAAGAC-3*`/*Rev 5`-TCGAGTTGTCCACAGTCAGC-3`*] and the mesenchymal marker Vimentin [*Fwd 5`-CGAAAACACCCTGCAATCTT-3`/Rev 5`-TCCAGCAGCTTCCTGTAGGT-3`*]. PCR Master Mix was prepared by mixing 5 μl cDNA together with 12.5 μl HotStarTaq Master Mix Kit (Qiagen, Cat. No. 203443, 250 U). Primers were added to a final concentration of 0.5 μM and the samples were filled up with RNase-free water to a total volume of 25 μl. Additional amplifications with 2 μl of the BreastDetect primer mix from the AdnaTest (BreastDetect, Adna Article no. T-1-509) were performed to analyse EpCAM, MUC-1 and Her2 gene expression. PCR was performed under the following conditions: after 15 min denaturation at 95°C, 42 cycles were carried out by denaturation at 94°C for 40 s, annealing/extension at 60°C for 40 s and elongation for 30 to 60 s at 72°C. A final elongation step at 72°C for 10 min was followed by storage of the samples at 4°C. The PCR generates products of the following sizes: Vimentin: 618 bp, EpCAM, MUC-1 and Her2 (as mentioned before), GAPDH: 138 bp, and PPIA: 133 bp. Visualisation was done with a Agilent 2100 Bioanalyser and peaks with a concentration above 1 ng/μl were defined as positive (Agilent Assay properties: normal). The actin primers of the AdnaTest could not be evaluated in this setting since they are not human-specific.

### Quantitative CTC detection - targeting human DNA (alu-sequences)

200 μl of murine blood were collected and red blood cells were lysed using RBC lysis solution from Qiagen for 10 min at 4°C (Cat. No. 158904). Next, samples were centrifuged for 10 min (450 g), followed by two washing steps using 1 ml of sterile PBS. DNA isolation was performed using DNeasy Mini Kits (Qiagen, Cat. No. 51306). 5 μl of the eluate were taken for qRT-PCR targeting human Alu-sequences [For 5`- CACCTGTAATCCCAGCACTTT-3`/Rev 5`-CCCAGGCTGGAGTGCAGT-3`] [13]. Primers were added to a final concentration of 0.5 μM and the samples were filled up with RNase free water to a total volume of 25 μl. PCR (Real-Time PCR System 7500, Applied Biosystems) using the QuantiTec SYBR Green PCR Kit (Qiagen, Cat. No. 204143) was done as follows: after 15 min denaturation at 95°C, 40 cycles were carried out by denaturation at 94°C for 60 s, annealing/extension at 60°C for 30 s and elongation for 30 at 72°C. Amplification curves were analysed and the number of CTCs were calculated by spiking experiments which were included in every run. DNA from murine blood and water were included as negative controls.

### Spiking experiments

Spiking experiments were done to test the sensitivity of the different methods. Fresh EDTA-blood was collected from female athymic NCr nude mice (*nu/nu*; Taconic). Cultured cells were harvested by trypsinisation and spiked into 1 ml of murine blood for the AdnaTest or 100 to 200 μl for detection of CTCs without antibody pre-enrichment. 1 to 50 cells were collected by pipetting under microscopic control, whereas higher numbers (1,000 and 100,000 cells) were prepared by dilution series. As negative controls, 20 murine blood samples were tested for each approach.

### Murine xenografts

5 to 6 week old female NCr nude mice were used throughout all xenograft studies. All experiments were approved by the local regulatory agency Landesamt für Gesundheit und Soziales Berlin (approval number: 1403). Mice were housed according to institutional guidelines with highest animal welfare standards [14]. For in vivo inoculation, harvested cells were counted with a CASY DT Cell Counter and Analyser (Innovatis AG). Cell number and viability were determined using the Analyser Software. Cells were kept at 4°C and resuspended in cold PBS or mixed with Matrigel (1 : 1) (BD Biosciences). 1 x 10^5^ to 2 x 10^6^ viable cells (MDA-MB-231, -468, and KPL-4) were injected orthotopically into mammary fat pads (m.f.p.) of the mice in a volume of 50 μl. Subsequently, mice were monitored for tumour growth.

Alternatively, for testing the stability of marker expression in CTCs, 1 x 10^5^ to 5 x 10^5^ cells of the KPL-4 and MDA-MB-231 line were injected into the tail vein or into the heart of female nude mice in a volume of 150 μl PBS.

### Blood collection and autopsy

We obtained EDTA-blood from either the jugular vein, the inferior vena cava or by cardiac puncture as tumours reached sizes between 20 to 200 mm^2^. Tumours, lymph nodes, lungs and livers were taken out and immediately frozen in liquid nitrogen for the detection of metastases by PCR of human transcripts.

### RNA isolation and mRNA amplification from tumours and tissues

Tissue was homogenised using Lysing Matrix D (MP Biomedicals, Cat. No. 116913500) and Fast Prep-24 System (MP Biomedicals, Cat. No. 116003500). RNA was purified using the RNeasy Mini Kit from Qiagen (Cat. no. 74104) and cDNA synthesis was performed with 400 ng of RNA input using the High Capacity RNA-to-cDNA Kit. PCR Master Mix was prepared as followed: 4 μl cDNA, 12.5 μl HotStarTaq Master Mix Kit and 2 μl of the primers from the AdnaTest kit or 0.5 μM in case of GAPDH, PPIA and vimentin. Each sample was filled up to 25 μl with RNase free water. Again, actin was excluded from analysis as signals were obtained in lysed tissues of tumour-free mice (data not shown). The Thermocycler was used for the PCR. Conditions were chosen as follows: 15 min denaturation at 95°C, 40 cycles of PCR were carried out by denaturation at 94°C for 40 s, annealing at 60°C for 40 s and elongation for 60 s at 72°C. Subsequently, termination of the reaction was carried out at 72°C for 10 min followed by storage of the samples at 4°C. 20 tumour-free (naive) mice were analysed as negative controls. Visualisation was done with an Agilent 2100 Bioanalyser and peaks with a concentration above 1 ng/μl were defined as positive (Assay properties set to “normal”).

### Gene expression analysis of CTCs

EMT-like changes on CTCs during tumour growth were analysed in orthotopic models using the “Human Epithelial to Mesenchymal Transition RT-Profiler PCR Array” System (SABiosciences, Cat. No: PAHS-090 C). This array profiles the expression of 84 genes that have been associated with EMT. 500 μl of blood were collected and transferred to RNAprotect Animal Blood Tubes. Total RNA was purified using the RNeasy Protect Animal Blood Kit and cDNA synthesis was performed using the RT^2^ First Strand Kit (SABiosciences, Cat. No. C-03/330401). A pre-amplification step was included using the PreAMP cDNA Synthesis Primer Mix (SABiosciences Cat. No. PBH-9090). Next, sample cocktails were loaded onto the array and amplification was performed as recommended by the vendor. Two murine blood samples were included as negative controls to test the species specificity of the assay. Although the array is intended to be human specific, only 14 genes could be included for data analysis in our study since mRNA signals for the remaining 70 genes were detected in blood samples from control mice. Genes with a cycle threshold (Ct) value below 36 in the control setting were excluded from expression analysis. As positive control, 10 cells of the KPL-4 cell line with epithelial phenotype were spiked into 500 μl of native blood. ΔCt values were calculated by subtracting the Ct value of the target gene from the Ct value of the house-keeping gene (GAPDH). ΔCt values of blood samples taken from tumour-bearing animals (KPL-4, n = 5) were subtracted from ΔCt values of the epithelial control (ΔΔCt). Negative ΔΔCt (relative quantification) values represent upregulated genes whereas positive values indicate downregulation of the markers.

## Results

### Detection of CTCs using the AdnaTest

**Figure 1:**
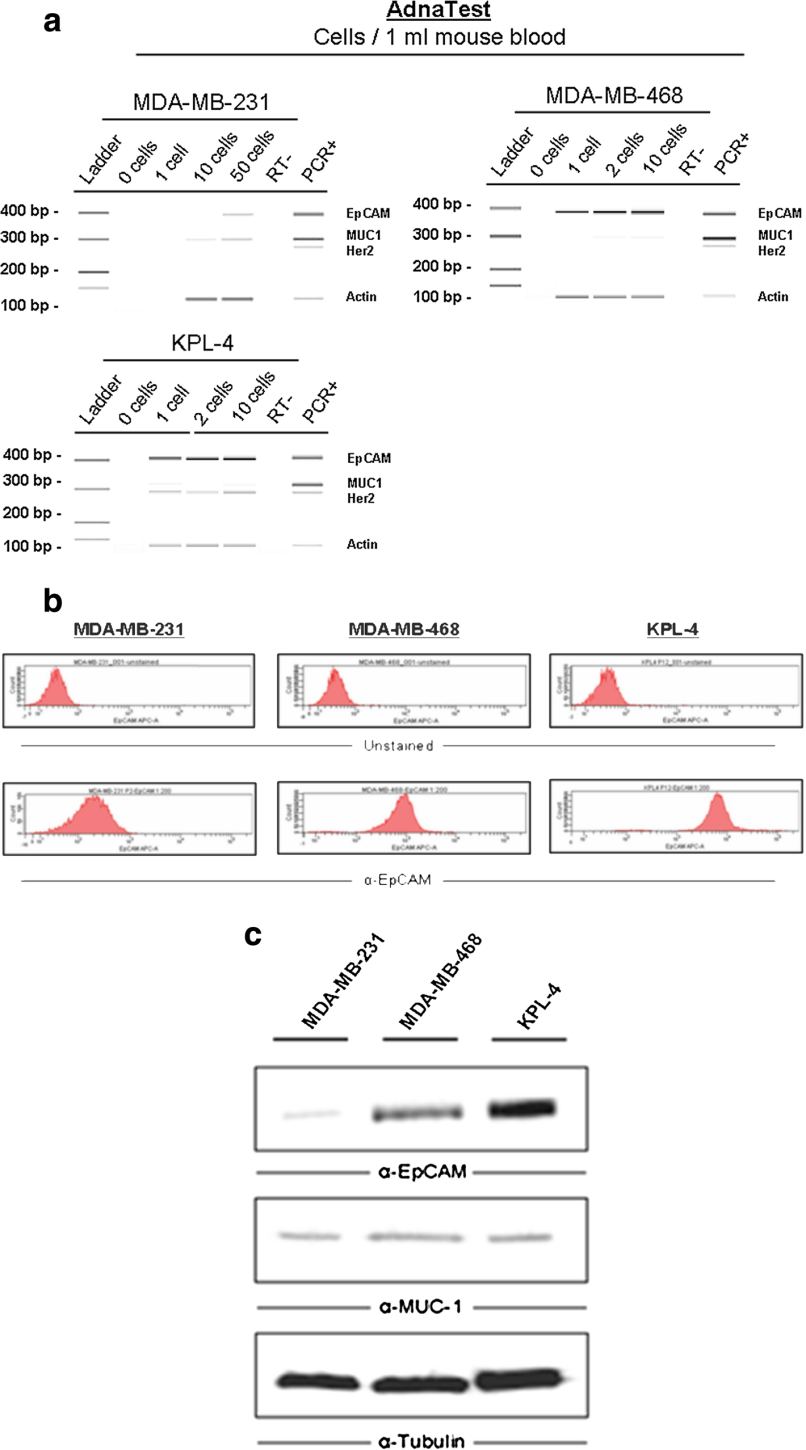
**a. Breast cancer cell detection by the AdnaTest - ex vivo.** Tumour cells were spiked into 1 ml of fresh mouse blood. Signals for human mRNA of EpCAM, MUC-1, Her2 and Actin were detected at different thresholds for each cell line. No false positive results were seen in control blood samples without human breast cancer cells. Negative controls without mRNA input were performed to exclude false positive results (“RT-“). Positive control was included in the AdnaTest Kit (“PCR+”). **b. FACS analysis for EpCAM expression.** Breast cancer cells were analysed by fluorescence activated cell sorting for EpCAM expression. Unstained cells are shown in the upper panels. Cells of the MDA-MB-231 line show very weak signals for EpCAM expression compared to MDA-MB-468 and especially cells of the KPL-4 line. **c. Western Blot analysis for EpCAM and MUC-1 expression .** Cells of the MDA-MB-231 line show weak signals for EpCAM expression compared to MDA-MB-468 and KPL-4 cells, whereas comparable MUC-1 expression levels are seen. Tubulin detection was performed as loading control.

To establish sensitivity and specificity of the AdnaTest System, 1 to 50 cells of human breast cancer cell lines MDA-MB-231, MDA-MB-468, and KPL-4 were spiked into 1 ml of blood collected from tumour-free mice. We could detect as few as 1 human cancer cell/1 ml blood when spiking cells of the MDA-MB-468 and KPL-4 line, which express high levels of EpCAM. In contrast, the detection limit for EpCAM^low^ cells (MDA-MB-231) was reached at 10 cells/ml (Figure 1a - c). We conclude that the detection limit of this assay is dependent on the EpCAM expression, since comparable MUC-1 RNA levels were detected in all three cell lines by a faint band in the samples containing 10 spiked cells (Figure 1a) and more relevant, similar protein expression levels were observed in all three cell lines (Figure 1c). Analysis of non-spiked blood samples revealed no positive signal, confirming the specificity of the assay.

**Figure 2:**
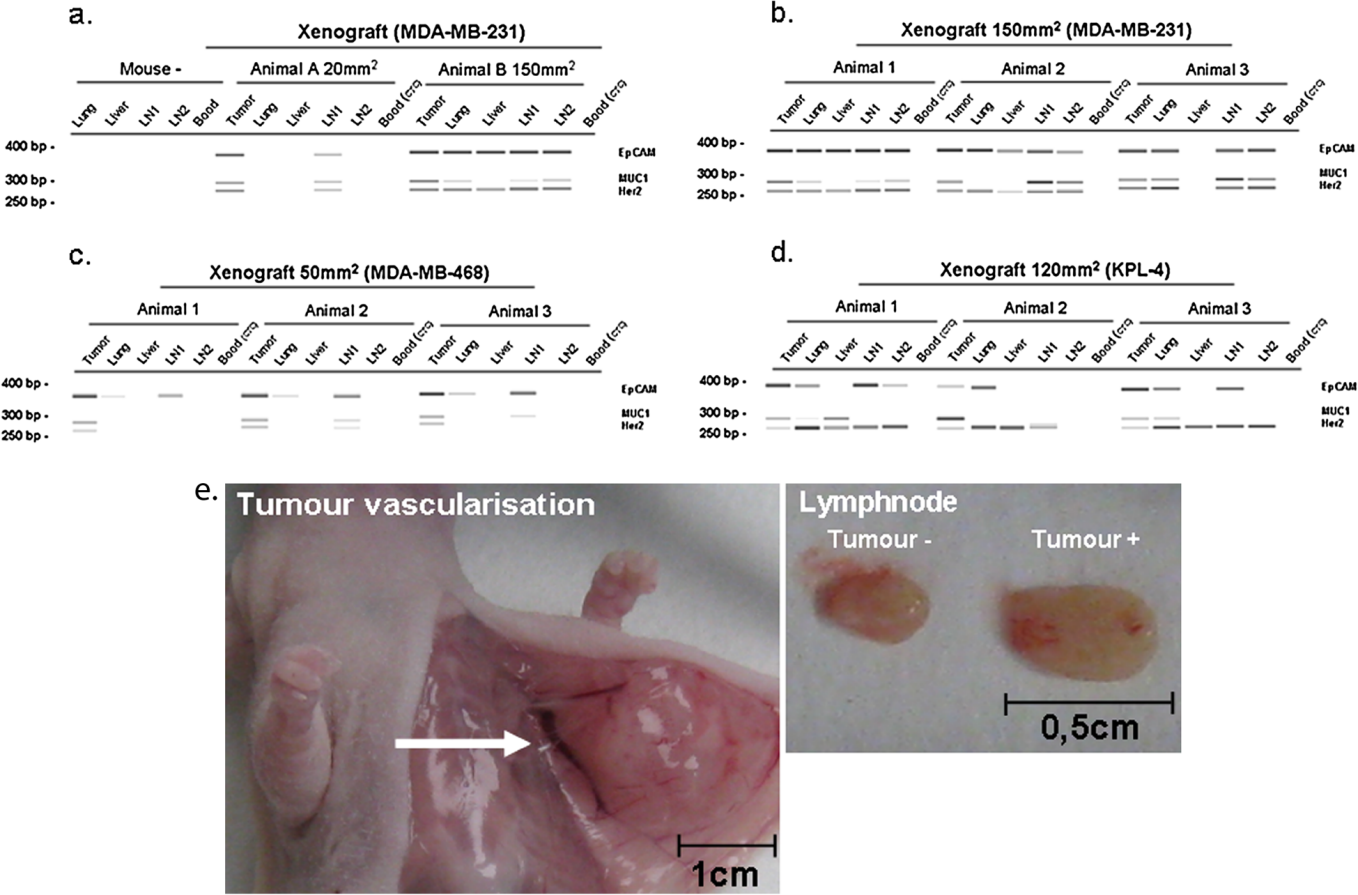
**Metastases and blood analysis of xenografted and tumour-free mice.** No human mRNA was detectable in tissue or blood of naive mice (a). Metastatic formation was seen in all xenografts. Most of the primary tumours and metastases were positive for mRNA of EpCAM, MUC-1 and Her2 expression but downregulation of EpCAM and mainly MUC-1 was observed, as well (a-d). No signals for human tumour cells could be detected by the AdnaTest in the blood of any murine xenograft although extensive tumour vascularisation and metastatic spread was seen in these mice (a-f).

Having validated the threshold, we next tried to detect CTCs in orthotopic breast cancer models (n = 96) bearing tumours of 20 to 200 mm^2^ in size (MDA-MB-231, MDA-MB-468 and KPL-4). To analyse possible metastatic spread, draining lymph nodes (*axillary:* LN1), remote lymph nodes (*inguinal:* LN2), lungs and livers were analysed for the presence of human mRNA. Such evidence for metastases was found in nearly all xenografted animals. The lymph nodes located next to the primary tumour or the lungs were infiltrated first during tumour growth (Figure 2a) and with increasing tumour size, metastases in livers and distant lymph nodes became visible as well. Most of the primary tumours and metastases were positive for EpCAM, MUC-1 and Her2 but in some cases, EpCAM and especially MUC-1 expression disappeared (Figure 2a - d). Despite extensive tumour vascularisation (Figure 2e) and metastatic spread, the AdnaTest system revealed no positive signal for CTCs in blood of any sample collected from jugular vein, inferior vena cava or by cardiac puncture (Figure 2a - d).

### CTC detection without pre-enrichment

We hypothesised that phenotypic changes associated with the epithelial-to-mesenchymal transition (EMT) and downregulation of the epithelial surface marker EpCAM could be responsible for our failure to detect CTCs using the AdnaTest. Therefore, we established two EpCAM-independent methods for CTC detection. The methods were based either on mRNA amplification of human gene transcripts (GAPDH, PPIA, EpCAM, MUC-1, Her2 and Vimentin) or amplification of human DNA (Alu-sequences).

**Figure 3:**
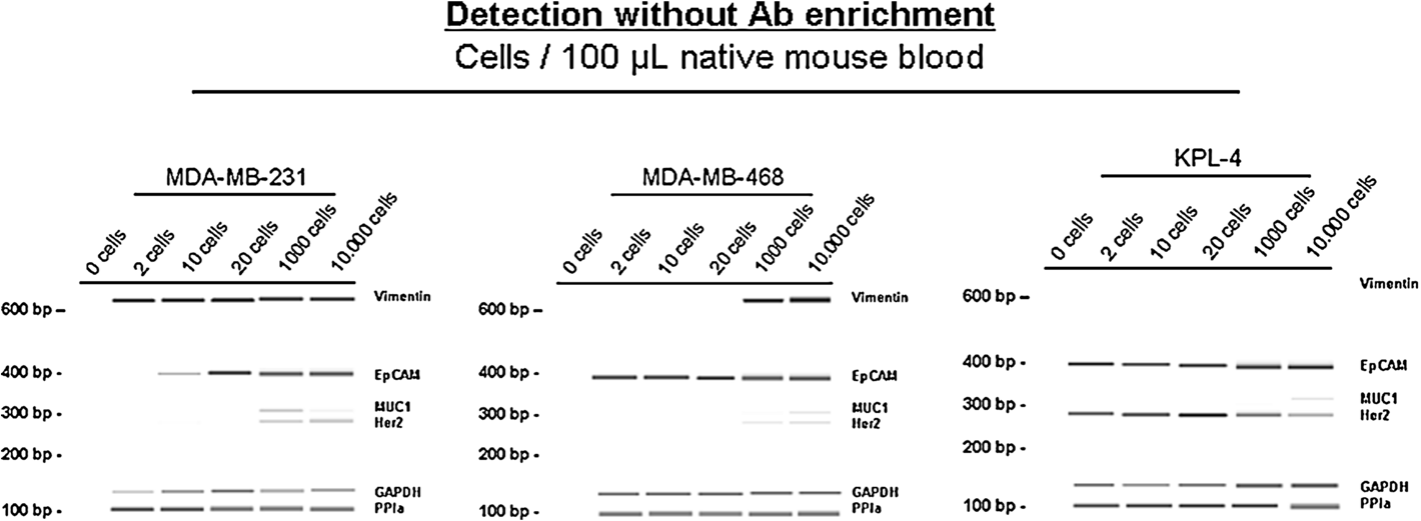
**Expression profile of breast cancer cells spiked into native mouse blood.** RNA was isolated and cDNA synthesis was performed. Human mRNA of Vimentin, EpCAM, MUC-1, Her2, GAPDH and PPIA was detectable at different cell numbers. No signals for murine mRNA were seen in native blood samples.

One to 10,000 human breast cancer cells (MDA-MB-231, MDA-MB-468 and KPL-4) were spiked into the blood of naive mice for assay validation. No false positive result was seen in blood from tumour-free control mice (n = 20), proving that the used PCR primers were specific to human sequences and therefore did not give any background signals, for example for Vimentin that would be expected in mesenchymal blood cells. All spiked samples showed positive signals for GAPDH and PPIA. As few as 2 tumour cells in 100 μl mouse blood could be reproducibly detected by expression of human housekeeping genes. EpCAM signals were detectable from 2 tumour cells or more for EpCAM^high^ cells (MDA-MB-468, KPL-4) but the detection limit was 10 tumour cells in case of EpCAM^low^ cells (MDA-MB-231) (Figure 3). Vimentin expression was detectable in cells of the basal like MDA-MB-231 line (2 cells/100 μl) and weakly in the MDA-MB-468 line (from 1,000 cells on) whereas no signals for Vimentin were seen in blood samples spiked with cells of the KPL-4 line (Figure 3). The cells also show the described Her2 expression pattern, very strong signals in KPL4 cells and very weak signals in MDA-MB-231 and MDA-MB-468.

**Figure 4:**
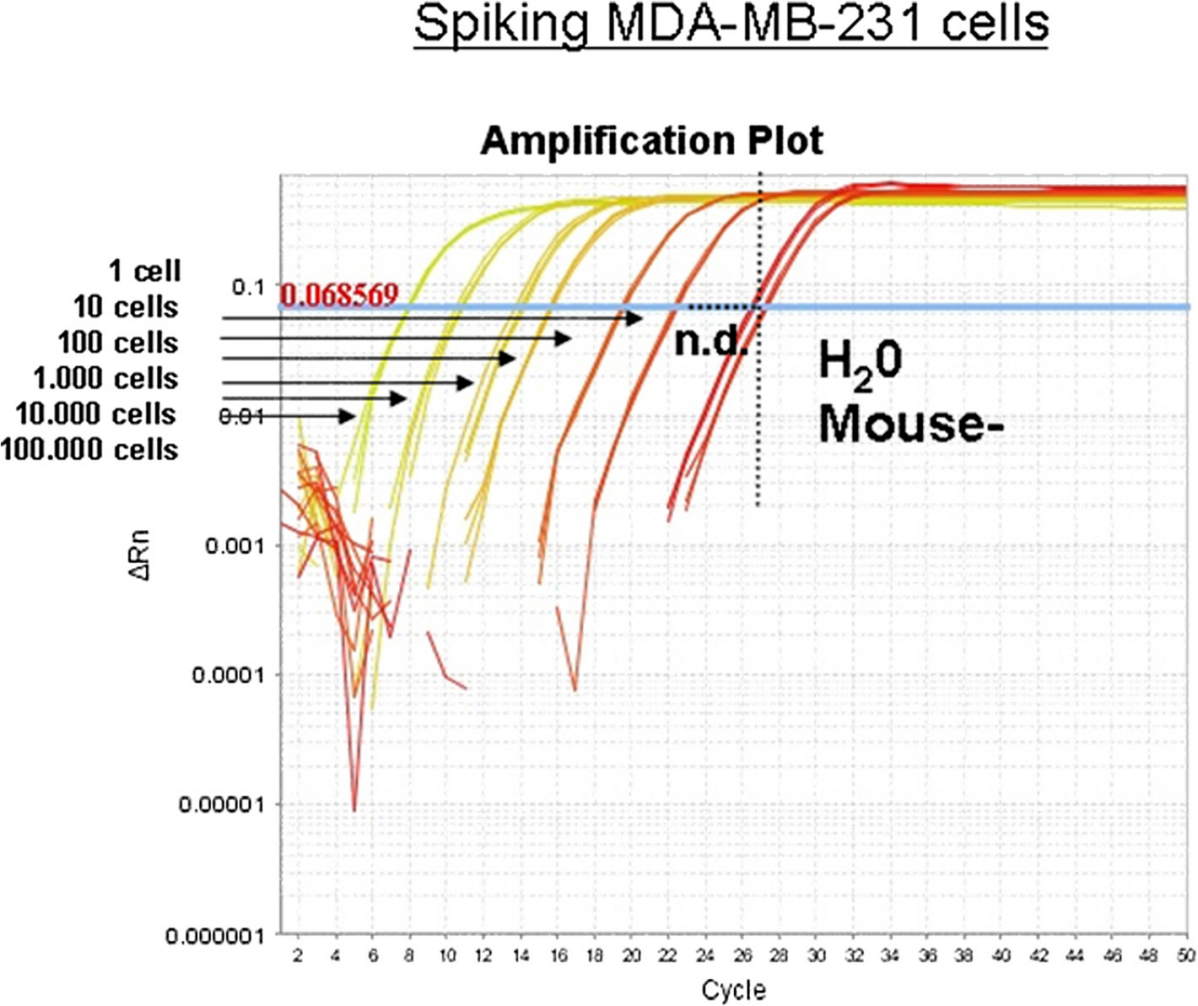
**Quantitative detection of CTCs targeting human Alu-sequences.** 1 to 100,000 human cancer cells were spiked into 200 μl of tumour-free murine blood. DNA was isolated and qRT-PCR was performed targeting human Alu-sequences. As little as 1 human cell could be detected before negative controls. Measurements were done in triplicates (n.d. = not defined).

For comparison, we also tested the detection of human cells by amplification of repetitive DNA sequences of the AluJ type. This approach could be more robust than testing RNA and should enable the quantification of cells due to the fixed copy number of these sequences. Using this approach, however, signals from 1 tumour cell in 200 μl blood could be detected in only 5 out of 8 (60%) spiking experiments (Figure 4). Signals from 10 cells or more were traceable in 100% of the analysed samples. Thus, the higher abundance of house-keeping gene mRNA leads to higher sensitivity of the RNA based CTC detection.

**Figure 5:**
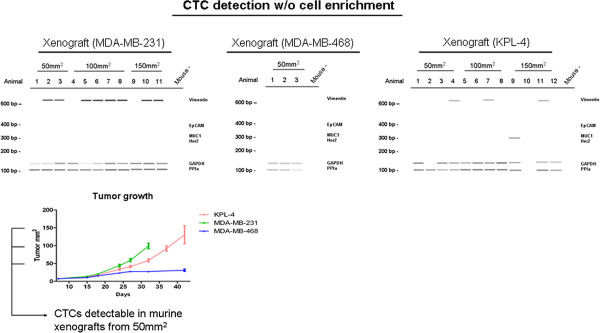
**CTC detection targeting human gene transcripts.** Representation of tumour growth in xenografted mice. CTCs were detectable at a tumour size of 50 mm^2^ for all tested cell lines. CTCs out of blood samples were mostly negative for mRNA of EpCAM, MUC-1 and Her2 but showed signals for housekeeping-genes and Vimentin (MDA-MB-231, KPL-4).

Having validated the methods, we next tested the performance of these CTC detection protocols in vivo. Using detection of GAPDH and PPIA transcripts, CTCs could be detected in nearly all blood samples collected by cardiac puncture (Figure 5). Positive signals were found in mice bearing tumours of 50 mm^2^ size, consistent with the appearance of lymph node metastases at this stage (Figure 2). In contrast, no signals for EpCAM, MUC-1 or Her2 transcripts could be detected in these samples, with the exception of one sample (Figure 5). Vimentin expression was, however, found in blood samples of mice bearing MDA-MB-231 tumours and surprisingly, in mice bearing xenografts from the KPL-4 cell line indicating EMT-like changes in this model, since no vimentin-specific transcripts could be detected in spiking experiments using cells taken directly from cell cultures (Figures 3 and 5).

Quantitative analysis revealed that blood samples contain CTCs at numbers which should be sufficient for detection with the AdnaTest, given that this assay detected as few as 1 cell/ml in spiking experiments (Table 1). Tumour cell numbers between 5 and 20 cells/ml (MDA-MB-231 and KPL-4) could be detected by targeting human Alu-sequences. However, no CTCs could be found when MDA-MB-231 cells were transplanted subcutaneously (Table 1).

**Table 1:** Quantitative detection of CTCs in tumour bearing animals

** Model**	**Tumour size**	**CTC**
**(Application)**	**(Animals)**	**(cells/ml)**
MDA-MB-231	20mm^2^	0/5
(orthotopic)	(5)	
MDA-MB-231	50 mm^2^	0/5
(orthotopic)	(5)	
MDA-MB-231	80 mm^2^	3/5
(orthotopic)	(5)	(5,10,and 15)
MDA-MB-231	120 mm^2^	1/5
(orthotopic)	(5)	(7)
MDA-MB-231	20-120 mm^2^	0/20
(subcutaneous)	(20)	
KPL-4	50 mm^2^	0/3
(orthotopic)	(3)	
KPL-4	80 mm^2^	1/2
(orthotopic)	(2)	(20)
KPL-4	120 mm^2^	1/4
(orthotopic)	(4)	(15)

CTCs could be quantified by qRT-PCR targeting human Alu-sequences in 6 out of 29 blood samples gained from xenografted animals. CTCs were detectable at tumour sizes between 80 to 140 mm^2^ (MDA-MB-231 and KPL-4). No CTCs could be detected in a subcutaneous MDA-MB-231 model. Number of CTCs were calculated by spiking rows which were included in every run.

### Phenotypic changes of CTCs after intravenous and intracardiac injection

**Figure 6:**
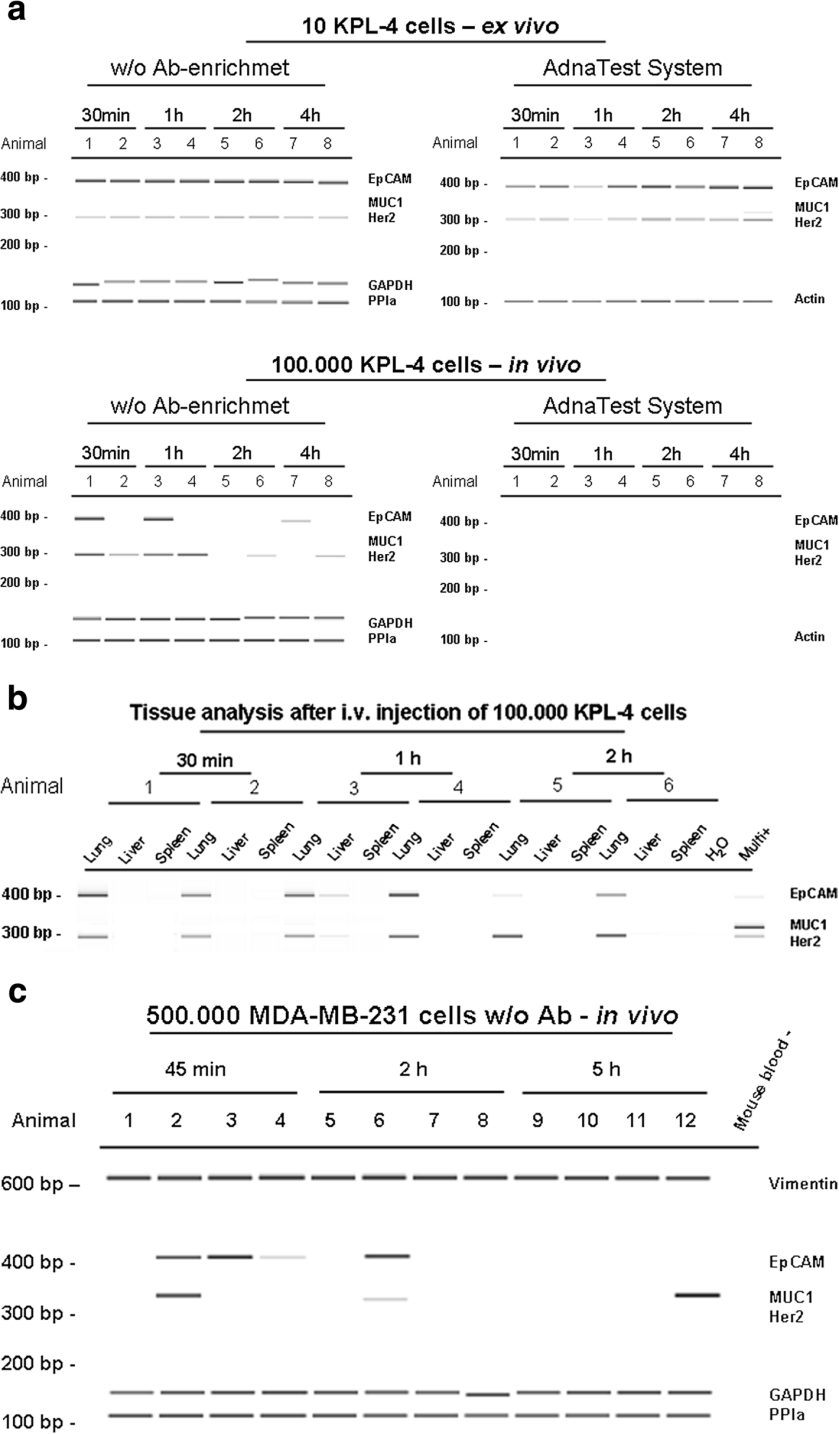
**a. Detection of spiked KPL-4 cells and cells after i.v. injection.** 10 cells were spiked into 100 μl or 1 ml of native murine blood samples. Cells of the KPL-4 line stably expressed EpCAM, Her2 and the housekeeping-genes for up to 4 h. The cells could be detected by AdnaTest and without antibody enrichment (a.). 100,000 cells of the KPL-4 cell line were injected into the tail vein of native mice. Tumour cells could not be detected by the AdnaTest, whereas human mRNA was visible without antibody enrichment for up to 4 h after injection. The detected cells showed serial changes and reduced EpCAM expression already 30 min after i.v. injection (b.). **b. Tissue analyses for human mRNA after injection of 100,000 KPL-4 cells into the tail vein.** Tissues were homogenised and RNA was purified followed by cDNA synthesis and PCR reaction. Human breast cancer cells detectable in the lungs and one liver were positive for EpCAM and Her2 expression up to 2 h after KPL-4 cell injection. **c. MDA-MB-231 breast cancer cells were injected into the heart and blood was analysed for human mRNA signals.** CTCs were detectable in all animals for up to 5 h after injection. The detected cells showed changes of mRNA expression including downregulation of EpCAM and MUC-1, whereas mRNA of the housekeeping-genes and mesenchymal Vimentin was visible permanently.

As we found a number of changes in gene expression comparing cell lines grown in vitro and in xenografts, we investigated the time course for changes in EpCAM and MUC-1 gene expression. First, we spiked 10 cells of the KPL-4 line into 100 μl or 1 ml of mouse blood. 30 min to 4 h later gene expression was assessed by qualitative PCR or the AdnaTest. Spiked cells were detected with both methods at all time points indicating stable mRNA and protein levels of EpCAM (ex vivo) (Figure 6a). Next, 100,000 cells of the KPL-4 line were injected into the tail vein of female mice and blood was collected by cardiac puncture for cell detection after 30 min to 4 h. No cells could be detected by the AdnaTest although 5 to 25 cells/ml were detected by amplification of human Alu-sequences (data not shown).

Tumour cells showed dynamic changes and down-regulated EpCAM and Her2 while circulating through the bloodstream (Figure 6a). Interestingly, cells adhering in the lungs kept EpCAM and Her2 mRNA expression (Figure 6b). To confirm these findings, we injected 500,000 cells of the MDA-MB-231 line into the heart of female mice. 45 min - 5 h after injection, blood was taken by cardiac puncture. Again, down-regulation of epithelial EpCAM and MUC-1 was observed, whereas transcripts of the mesenchymal marker vimentin and the house-keeping genes were detected in 100% of the blood samples (Figure 6c).

**Figure 7:**
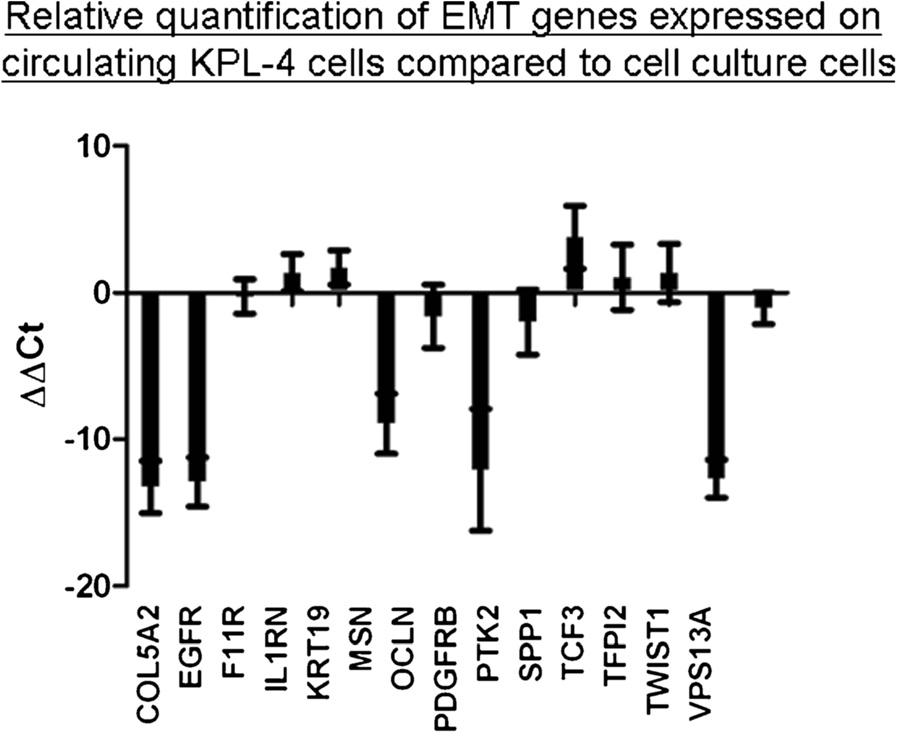
**Profile changes of in vitro KPL-4 cells compared to circulating KPL-4 cells out of tumour bearing animals.** Samples were analysed using RT^2^ Profiler PCR Array System focusing on EMT specific genes. Data analyses shows that COL5A2, EGFR, MSN, PDGFRB and Twist were upregulated on CTCs whereas downregulation of CK-19 and SPP1 could be observed (n = 5).

A more detailed comparative analysis of KPL-4 cells taken from cell culture and circulating KPL-4 cells in blood from tumour-bearing mice revealed changes in expression of EMT-associated genes with upregulation of Twist, Collagen-alpha-2(V)-chain (COL5A2), epidermal growth factor receptor (EGFR), moesin (MSN) and beta-type platelet-derived growth factor receptor (PDGFRB) in the latter. In contrast, expression of epithelial markers like cytokeratin-19 (CK-19) and osteopontin (SPP1) was slightly reduced in CTCs (Figure 7).

## Discussion

We confirm here previous reports that the AdnaTest is a very sensitive assay for detection of epithelial cells. We also demonstrate that the sensitivity of the assay was significantly dependent on the expression levels of EpCAM, which was causally linked to its failure to detect any positive CTC sample in murine breast cancer xenograft models. We were able to show that EMT occurs in vivo during formation of metastases in tumour-bearing mice. These changes include the down-regulation of EpCAM. We showed for the first time that gene expression changes in CTCs are very fast and seem to depend on contact with endothelial surfaces or organ systems in vivo, since even i.v. injected tumour cells quickly down-regulated EpCAM. The surprisingly fast loss of EpCAM could be explained by cleavage and shedding of the extracellular domain as described [15,16]. Besides the down-regulation of EpCAM, we identified up-regulation of mesenchymal markers like COL5A2, EGFR, MSN, PDGFRB and Twist in CTCs, supporting the epithelial-to-mesenchymal transition as the cause for down-regulation of EpCAM. Confirming our findings, COL52A has been identified as marker for EMT, since TGFβ/TNFα-treatment induced a mesenchymal cell phenotype accompanied with increased expression of this gene [17]. EGF signaling has also been previously linked to tumour progression and metastasis [18-21]. We found dramatically increased EGFR expression in CTCs, confirming the role of this pathway in the process of metastatic spread. It has been shown that EGF can induce EMT-like effects including upregulation of Twist through the EGFR pathway [22]. Twist expression has been described as a crucial regulator of EMT and metastasis formation [23]. CTCs also showed elevated Twist expression levels in our model, confirming these findings [23]. The modified gene expression profile that we report here could provide the basis for alternative methods for isolation and detection of CTCs from clinical samples. The surface marker EGFR may be useful for capturing mesenchymal-like CTC subpopulations, whereas the intracellular proteins like vimentin, collagen-alpha-2 and Twist could be used for improved detection of CTCs by immunostaining. Such approaches will be especially useful to confirm the identity of CTC populations isolated by size exclusion as recently described [24]. The isolation of CTCs based on their size is independent of epithelial marker expression and therefore leads to higher CTC yields. A larger set of tumor-specific markers will be useful in confirming the identity of CTC populations enriched by filtration methods.

In contrast to this study, CTC detection has previously been described using an EpCAM-based method in a murine xenograft model [9]. In addition, CTC-positive samples were found in murine prostate cancer xenografts using a CTC-chip [10], which has been previously described and evaluated [25]. Either EpCAM expression was not downregulated in the PC3 model or the CTC chip captures even cells with very little residual EpCAM expression. This hypothesis is supported by the fact that low EpCAM cells are captured late on the CTC-chip whereas cells with high EpCAM expression are bound already in the beginning of the flowpath (pers. communication S. Nagrath). Down-regulation of MUC-1 and Her2 on CTCs has also been noted by this group, in keeping with our findings. More recently, increased vimentin expression, indicative of EMT processes in CTCs in another orthotopic breast cancer model, has been published [26]. Taken together, these results demonstrate that phenotypic changes occur in CTCs. For this reason, we believe that new methods are needed to ensure that the complete phenotypic variability of CTC populations can be described.

Although the prognostic value of CTC counts have been demonstrated using epithelial marker based CTC detection methods [27-30], our findings indicate that numerous cells may be lost in many blood samples of cancer patients due to EMT processes. This may explain why even in late stage metastatic patients, only around 50% of the patients with breast or prostate cancer have more than 5 CTCs and in case of colorectal cancer only 26% more than 3 CTCs in 7.5 ml blood. Other authors failed to detect subtypes of CTCs resembling normal type breast cancer using the CellSearch technique [31]. Further, significantly higher numbers of CTCs could be detected using an EpCAM independent detection method compared to EpCAM based enrichment technology (69.2% vs. 42.3%) in breast cancer patients [32], suggesting that a mixture of EpCAM positive and EpCAM negative tumour cells circulate in the blood. In a study of neuroendocrine cancer patients using CellSearch, CTCs were found in only 21-43% of the patients, although 100% of the tumours showed strong membrane EpCAM expression by IHC [33]. Down-regulation of EpCAM has been reported independently for DTCs in bone marrow and CTCs in peripheral blood of patients with breast cancer and for other solid tumors [34,35]. These publications demonstrate that the use of anti-EpCAM antibody-based enrichment strategies limits the clinical utility the CellSearch System, the AdnaTest or other EpCAM based procedures. Our own data and several recent studies support the hypothesis that EMT-like shifts on CTCs occur during metastatic spread [5-7,36]. These results indicate that CTCs will be lost using immuno-affinity enrichment that relies only on the level of EpCAM expression on the cell surface [37].

Taken together, our findings explain low CTC numbers in clinical studies even in metastatic cancer. The use of EpCAM-based enrichment techniques leads to the selective loss of CTC populations undergoing epithelial-to-mesenchymal transition. We could show that new technologies have to be developed which securely isolate all CTCs. Methods for the analysis of CTCs in clinical samples should be designed to detect both epithelial and mesenchymal-like subpopulations. Our findings could be used as a starting point for the qualification of new markers for CTC detection or capture including the mesenchymal-like subpopulations. Future studies should consider the heterogeneity of CTC populations and find out which one is prognostic for the course of the disease and which population may give best hints towards therapeutic options.

## Conclusion

As we have shown in this work, the use of single surface markers for isolation of CTCs can lead to the loss of CTC populations having undergone phenotypic changes like the epithelial to mesenchymal transition. This finding may explain the clinical results with low CTC counts or even absence of detectable CTCs in many even late metastatic cancer patients. Future research has to identify new selection markers that are robustly expressed and not subject to phenotypic changes. An alternative is the enrichment of CTCs based on size and a detailed molecular analysis of the enriched cell population especially in respect to predictive markers like target expression and/or specific mutations.

## Competing interests

All authors, except Ingeborg Tinhofer are full time employees of Bayer Pharma AG and hold company shares.

## Authors’ contributions

TG: Performed all experiments except FACS analysis. IT: Manuscript writing and scientific discussion. MD: FACS analysis and scientific discussion. LR: Manuscript writing and scientific discussion. TMZ: Administration, funding and scientific discussion. TK: Administration, funding and scientific discussion. OA: Supervision and manuscript writing. All authors read and approved the final manuscript.

## Pre-publication history

The pre-publication history for this paper can be accessed here:

http://www.biomedcentral.com/1471-2407/12/178/prepub
